# Jaw Pain as a First Presentation in the Diagnosis of Breast Cancer

**Published:** 2016-12-24

**Authors:** Payam Azadeh, Nasser Rakhashani, Ali Yaghobi Joybari, Pegah Gorji Bayani, Samaneh Sarbaz, Maryam Farasatinasab

**Affiliations:** 1 *Dept. of Radiation Oncology, Shahid Beheshti University of Medical Sciences, Tehran, Iran*; 2 *Dept. of Pathology, GILDRC,* * Firoozgar Hospital, Iran University of Medical Sciences, Tehran, Iran*; 3 *Det. of Clinical Pharmacy, School of Pharmacy-International Campus, Endocrine Research Center, Institute of Endocrinology and Metabolism, Firoozgar Hospital, Iran University of Medical Sciences, Tehran, Iran*

**Keywords:** Breast Cancer, Jaw Pain, Odontogenic Fibroma, Mandibular Metastasis

## Abstract

The oral cavity is uncommon site for metastatic disease usually discovered secondary to malignancy. We encountered with a rare case in which metastasis to mandibular bone was the first clinical sign in the diagnosis of breast cancer without any radiographic findings. A 49-yr-old premenopausal woman, was referred to the Department of Medical Oncology of Imam Hossein Hospital, Shahid Beheshti University of Medical Sciences, Tehran, Iran in 2014, presented with pain and tenderness in the left mandibular and temporal bone and paresthesia of the lower left lip and chin. CT scan of mandible showed no significant finding. Four months later, she was referred with complaints left breast pain for 4 wk and worsening swelling, pain and paresthesia. Breast examination revealed a 2 cm firm nodule on the left breast. Based on her medical history and histopathological study, metastatic carcinoma of the breast was suspected. She has received chemoradiotherapy that led to complete relief of her symptoms and remission of the disease. In the presence of an ambiguous sign in oral cavity such as jaw pain or paresthesia, diagnostic examination of malignancy is recommended.

## Introduction

The oral cavity is rare site for metastatic dissemination, accounting for only 1% of all malignancies in the region (1). This involvement is usually diagnosed secondary to malignancy; however, in approximately one-third of cases metastasis is the first clinical manifestation (1- 3). “The most common primary origins include the lung, kidney, liver, and prostate for men and breast, female genital organs, kidney, and colorectum for women” (4); however, primary site could be affected by geographic location (4- 7). In the most of cases, radiographic appearance increases the suspicion of malignancy; but pathological changes are not observed in about 5% of the radiographs (3). The most common sites for breast cancer to metastasize are the bone, lung, liver, lymph nodes, and brain (8). 

We encountered with a rare case in which metastasis to mandibular bone was the first clinical sign in the diagnosis of breast cancer without any radiographic findings, highlighting the importance of clinical manifestation in the oral cavity and incisional biopsy and immunohistochemical techniques in the diagnosis.

## Case report

A 49-year-old premenopausal woman presented with complaints of pain and tenderness in the left mandibular region and paresthesia of the lower left lip and chin in Jul 2014. She had denied any medical, surgical, or significant family history. Her last mammogram was performed 6 months ago for screening, which was unremarkable. A CT scan of the head and neck revealed no significant finding. She was started on a trial of gabapentin, duloxetine, and carbamazepine for trigeminal neuralgia. In Dec 2014, she was referred to the Department of Medical Oncology of Imam Hossein Hospital, Shahid Beheshti University of Medical Sciences, Tehran, Iran with complaint of pain and tenderness over the left breast and worsening of pain and paresthesia the left half of the face that did not respond to any analgesic medication. Breast examination revealed a 2-cm firm nodule on the left breast. Informed consent was taken from the patient.

**Fig. 1:**
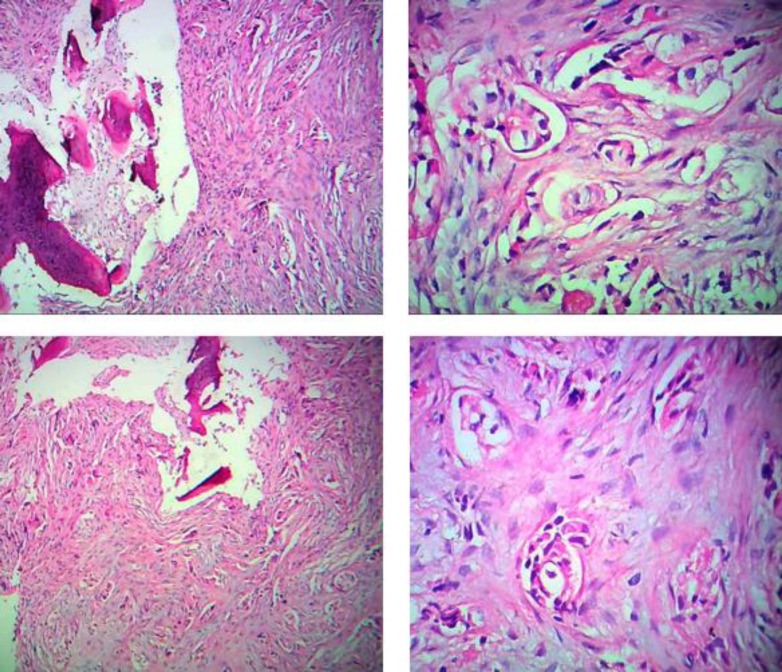
Malignant ducts of breast origin infiltrating jaw bone

Her pathologic report was consistent with invasive ductal carcinoma (IDC) of two left breast mass and axillary lymph node. Immunostaining was positive for ER/PR but negative for HER-2; Ki67 was positive in approximately 40% of cells. A whole body bone scan (WBBS) revealed abnormal isotope accumulation in the left mandible, suggestive of bony infiltration. Consequently, CT scan of the head and neck was performed which revealed thickening and sclerosis of the body and ramus of the left hemimandible. Incisional biopsies were obtained and composed of small needlelike firm tissue with bone consistency measuring 1 cm in length and 0.4 cm in width, showing a neoplasm not related to the mandibular region, made up of hyperchromatic nuclei and large eosinophilic cytoplasm cells. The cells were disposed of in abortive glandular-like structures with significant sclerosis placed between segments of bone and into bone marrow spaces. The tumor cells exhibited positive immunostaining for CK7, ER/PR, GCDFP-15, and Mammaglobin and negativity for CK20. These histological findings were suggestive of metastatic invasive ductal carcinoma (Fig. 1). Other metastatic workup, including spiral CT scan of the thorax, abdomen, and pelvic area, were normal. She was started on doxorubicin/cyclophosphamide/5-FU chemotherapyy, and local irradiation of the left mandible was performed. The patient underwent radiation therapy over 2 wk, which resulted in relief of her symptoms and remission of the disease.

## Discussion

The oral cavity is uncommon site for metastatic disease reported only approximately 1% of all oral malignancies (1). The primary tumor is usually known before the oral metastatic lesion appeared in most patients (1- 3). In a study, only 169 cases with the oral metastatic tumor, the first sign was involvement this region (3). The most primary site of metastatic lesions is in diminishing order the breast, lung, kidney, and prostate; although it appears that geographic location mainly between east and west may be effect of region involvement (4). The uterus was the most common primary location in women (6). Furthermore, the liver and thyroid was recognized to be the most common main sites for men and women, respectively (5).

Oral metastatic lesions may occur into mucosal and jawbone, however, the jawbones, especially mandible are more frequent for metastasis than the oral mucosa (4). Swelling, pain and paraesthesia are the common symptoms of a metastatic tumor in the jawbones. Mental nerve neuropathy, known as ‘‘numb chin syndrome’’ could be the sign of a metastatic disease in the mandible; however, these symptoms could result from trauma, infection or noticeable odontogenic reasons and systemic diseases such as amyloidosis, sarcoidosis, or as neurological manifestation of a non-metastatic malignancy (9). Therefore, early detection of jawbone metastasis is usually difficult. In the early stages, lesions may not cause radiological features (3, 10) and the pathological evaluation of the lesions is mandatory for diagnosis. In our report, mandibular involvement was first sign of malignancy that the computed tomography failed to detect it in early stages.

 The most common histopathological types of primary tumors including breast are adenocarcinoma (1, 11). However, ductal carcinoma, such as our case, may be observed in breast involvement (11). Likewise, to confirm the primary site of tumor immunohistochemical techniques is necessary. Breast neoplasm usually present CK7 but not CK20 (4) and along with positivity for ER/PR, GCDFP-15, Mammaglobin could be ruled out other malignancy. 

The mechanism of metastatic tumors to the oral cavity is unknown, but a hematogenous spread from a distant region is considered. In the breast cancer, the bone, lung, liver, lymph nodes, and brain are the most common sites for metastasis, but oral cavity involvement is very rare (1-4, 11). 

Palliative therapy is primarily management of metastatic breast cancer to the oral cavity and generally includes chemotherapy, hormone therapy and local radiotherapy (12). Oral metastasis carries a poor prognosis for the patient because it represents advanced disease; however, in this case, primary tumor presentation can be a solitary mandibular metastasis (10).

Manifestations of malignancy including breast are not always straightforward. Therefore, in the presence of an ambiguous sign in the oral cavity, such as jaw pain or paresthesia, a differential diagnosis must include metastatic dissemination, and diagnostic examination is highly recommended.

## Conflict of Interests

The authors declare that there is no Conflict of Interests. 
